# Gastric Perforation: A Rare Accident from Intubation

**DOI:** 10.7759/cureus.6684

**Published:** 2020-01-16

**Authors:** Kulachanya Suwanwongse, Nehad Shabarek

**Affiliations:** 1 Internal Medicine, Lincoln Medical Center, New York City, USA

**Keywords:** intubation, complication, gastric perforation, gastric rupture, airway

## Abstract

Perforation of a gastrointestinal tract as a complication of intubation is unusual, and only few cases have been reported. Prompt recognition and management of gastrointestinal tract perforation are needed to limit the morbidity and mortality of this condition. We presented a case of an acutely ill patient who developed gastric perforation following difficult intubation to remind clinicians of a life-threatening complication that can develop following a life-saving procedure.

## Introduction

Perforation of the stomach is an urgent surgical condition, which requires prompt diagnosis and treatment to avoid fatal outcomes. Untreated gastric perforation can lead to peritonitis, septic shock, and death. Peptic ulcer disease is the most common cause of gastric perforation, whereas iatrogenic gastric perforation is uncommon and mainly occurs during operation, cardiopulmonary resuscitation, or gastric tube placement [1]. While conservative management may be used in the selected cases of gastric ulcer perforation, surgery remains the primary management for iatrogenic gastric perforation and should be done emergently to limit patients' morbidity and mortality. Difficult intubation resulting in accidentally esophageal intubations can lead to several tragic complications, including the rupture of organs. We reported a rare case of iatrogenic gastric perforation following difficult emergency intubation to raise physicians’ awareness of an unusual life-threatening complication from a common life-saving procedure. 

## Case presentation

**Figure 1:**
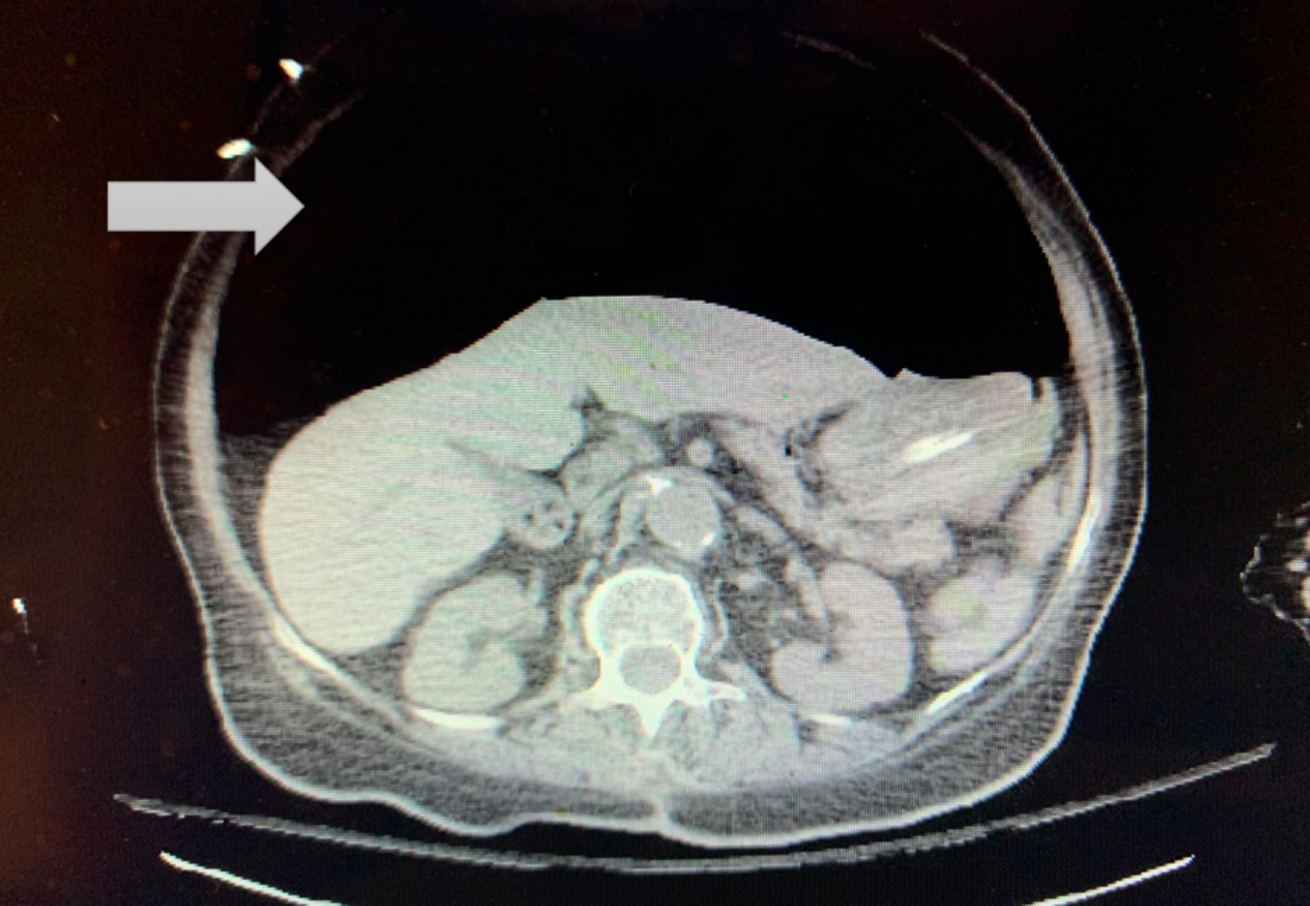
CT scan of abdomen shows massive pneumoperitoneum

A 76-year-old woman presented to our emergency department with severe headache, vomiting, and alteration of mental status. She had a past medical history of cerebrovascular disease with left-sided residual weakness, hypertension, type 2 diabetes mellitus, and hyperlipidemia, but did not report any previous history of dyspepsia nor peptic ulcer diseases. The decision was made to intubate her for airway protection due to decreased levels of consciousness. She had failed endotracheal intubation from esophageal intubation twice. After successful intubation performed by an anesthesiologist, she was found to have significant abdominal distension. The orogastric tube was placed to suction, but no significant decompression was achieved. Pan computed tomography (brain, chest, abdomen, and pelvis) identified massive intracranial hemorrhage with obstructive hydrocephalus and very large pneumoperitoneum, as demonstrated in Figure 1. She underwent exploratory laparotomy, which found 9 cm perforation of the anterior gastric wall and serosal tear in the posterior gastric wall; all were repaired with silk sutures. Neurosurgery placed right-sided external ventricular drainage for hydrocephalus but decided not to perform any other decompression surgery due to grave prognosis. She did not have any acute complications from exploratory laparotomy but progressed to brain death due to massive hemorrhagic stroke. The patient expired shortly after palliative extubation.

## Discussion

Iatrogenic gastric perforation is a life-threatening complication that carries significant morbidity and mortality, requiring urgent surgical attention. Gastric perforation should always be suspected if patients develop a sudden increase in abdominal distention or a tense abdomen following difficult intubation. The most common location of gastric perforation is at the lesser curvature of the stomach because this area is less elastic, has fewer mucosal folds, and relatively fix to hepatogastric ligaments [2]. Air and gastric content are released to the peritoneal space, following the ruptures of the stomach. This leads to chemical peritonitis and, later on, bacterial peritonitis. Untreated gastric perforation can result in shock and death.

Our patient had no prior history of peptic ulcer diseases nor chronic non-steroidal anti-inflammatory drug use. She had unintentional esophageal intubation twice and received Ambu-bagging in between, which most likely contribute to a gastric perforation due to an overinflate stomach. In general, the stomach of a healthy adult individual can tolerate pressure up to 120-150 mmHg before its rupture [3]. Abdominal distention and generalized peritonitis signs, including rebound tenderness and rigidity, are commonly found following the rupture of the stomach because of the release of gastric content and air. Upright chest X-ray may identify air under the diaphragm. Decompression of the stomach, including aspirating of gastric contents and air via a gastric tube, will not resolve abdominal distention following gastric perforation, as observed in our patient. The reason for unresolving distention is that the air and fluid are already released into the peritoneal space.

The standard treatment for patients with iatrogenic gastric perforation is laparotomy with primary closure of the rupture sites, peritoneal lavage, and systemic antibiotics. Prevention of gastric perforation following a failed endotracheal intubation should start with the identification of the patient at risk for difficult intubation and the use of good techniques and practices [4]. Early identification of accidental esophageal perforation is needed to prevent gastric wall tension and tear from controlled ventilation. Prompt recognition of iatrogenic gastric perforation will also help in reducing the morbidity and mortality in general.

## Conclusions

We presented an interesting case of gastric perforation in an acutely ill elderly, who required emergent airway management. Although gastric perforation is a rare complication of intubation, clinicians should have suspicious of this fetal complication particularly in patients with difficult intubation, esophageal intubation, having abdominal distention, tenderness, or guarding after intubation. 
